# CD4^+^ T Cell Defects in a Mulibrey Patient With Specific *TRIM37* Mutations

**DOI:** 10.3389/fimmu.2020.01742

**Published:** 2020-09-18

**Authors:** Sara Bruzzaniti, Emilia Cirillo, Rosaria Prencipe, Giuliana Giardino, Maria Teresa Lepore, Federica Garziano, Francesco Perna, Claudio Procaccini, Luigi Mascolo, Cristina Pagano, Valentina Fattorusso, Enza Mozzillo, Maurizio Bifulco, Giuseppe Matarese, Adriana Franzese, Claudio Pignata, Mario Galgani

**Affiliations:** ^1^Laboratorio di Immunologia, Istituto per l'Endocrinologia e l'Oncologia Sperimentale “G. Salvatore”, Consiglio Nazionale delle Ricerche, Naples, Italy; ^2^Dipartimento di Biologia, Università degli Studi di Napoli “Federico II”, Naples, Italy; ^3^Dipartimento di Scienze Mediche Traslazionali, Università degli Studi di Napoli “Federico II”, Naples, Italy; ^4^Unità di Neuroimmunologia, Fondazione Santa Lucia, Rome, Italy; ^5^Dipartimento di Medicina Clinica e Chirurgia, Università degli Studi di Napoli “Federico II”, Naples, Italy; ^6^Divisione di Farmacologia, Dipartimento di Neuroscienze e Scienze Riproduttive ed Odontostomatologiche, Università degli Studi di Napoli “Federico II”, Naples, Italy; ^7^Dipartimento di Medicina Molecolare e Biotecnologie Mediche, Università degli Studi di Napoli “Federico II”, Naples, Italy

**Keywords:** immune response, CD4^+^ T cells, immunological defects, TRIM37, Mulibrey syndrome

## Abstract

Mulibrey (muscle-liver-brain-eye) syndrome (MUL) is an autosomal recessive disorder caused by mutations in the *TRIpartite motif* (*TRIM*)*37* gene, encoding for TRIM37 a member of the TRIM E3 ubiquitin ligase protein family. MUL patients are characterized by growth retardation, dysmorphic features, and a wide range of abnormalities affecting different organs. However, T-cell abnormalities have not been observed in MUL subjects, to date. Here we described the immunological features of a MUL child carrying recently identified *TRIM37* mutations, a 17q22 deletion of maternal origin combined with a *TRIM37* variant of paternal origin. Here we found quantitative and functional defects in CD4^+^ T cells from this MUL case. Low levels of TRIM37 protein were specifically detected in CD4^+^ T cells of MUL patient and associated with their altered proliferation and cytokine production. Of note, both CD4^+^ and CD8^+^ T lymphocytes of MUL child displayed an effector memory phenotype compared with healthy children. This clinical case research highlighted the possible role of TRIM37 in the control of immune cell number and function, especially in CD4^+^ T cells. Finally, this study may contribute to the novel mechanistic studies aim of identifying, in depth, the role of the TRIM37 protein in the immune system.

## Introduction

Mulibrey (muscle-liver-brain-eye) nanism (MUL) is a rare autosomal recessive growth disorder, caused by mutations of *TRIpartite motif* (*TRIM*)*37* gene, located on chromosome 17q22-23. Human *TRIM37* contains 25 exons, and TRIM37a (its main human transcript) contains 4.33 kb and encodes a 964 amino-acids protein expressed in several tissues (1). To date, about 25 *TRIM37* mutations with different genomic localization and/or geographical origin have been identified (2). The so-called “Fin-major mutation” is a c.493-2A>G transition in the 3' splice site of exon 7, leading to a premature stop codon and a truncated protein of 174 amino-acids. Intragenic rearrangements and gene deletions have also been reported in non-Finnish MUL patients (3, 4). Clinically, MUL subjects are characterized by severe pre- and post-natal defects. Fibrosis and constrictive pericarditis are the most serious abnormalities of MUL syndrome and are present in the 20% of the patients (5). Type 2 diabetes, fatty liver, and hypertension are also associated with the disease (2). Furthermore, MUL children display a high frequency of both benign and malignant tumors in different organs (4, 5).

TRIM37 is a member of the TRIM superfamily proteins, characterized by a RING type E3 ubiquitin ligase activity (6). As ubiquitin ligase, TRIM proteins mediate the transfer of ubiquitin to substrate target proteins and are involved in many biological processes, including post-translational modifications, signal transduction, DNA repair, immunological signaling, autophagy, and oncogenesis (7).

Protein ubiquitination represents a crucial process in the immune system and the association between several TRIM proteins with T cell signaling pathways (8) supported the hypothesis that TRIM37 can be involved in the control of immune responses. Accordingly, Haraldsson et al. revealed humoral immunodeficiency in a patient affected by MUL syndrome (9). However, none of the published evidence reported adaptive immune response defects in MUL individuals (5, 10).

Here we analyzed immunological alterations in a MUL child with recently identified *TRIM37* genetic mutations consisting of a 17q22 deletion of maternal origin and a *TRIM37* variant (c.1949-12A>G in intron 18) of paternal origin, causing a new acceptor splice site and the introduction of a premature stop codon (4).

In this patient, we found a specific reduction of TRIM37 protein expression in CD4^+^ T cells. This finding is associated with a selective impairment in the number and function of the CD4^+^ T cell subset. Moreover, both peripheral CD4^+^ and CD8^+^ T lymphocytes from the MUL child showed an unusual memory-like phenotype (11). Our findings are consistent with an overall scenario of T cell defects associated with *TRIM37* mutations, thus opening a new line of research to explore in depth the role of TRIM37 in immune response.

## Results

### Case Presentation

The MUL patient is an 11-year-old boy born from unrelated Caucasian parents, with no familiar history of primary immunodeficiency disorders. As previously described in Mozzillo et al., clinical phenotype was characterized by intrauterine growth retardation, facial dysmorphic features with relative macrocephaly (head circumference SDS>1.5 population mean for age), skeletal abnormalities, and severe postnatal growth retardation (height SDS < -2 population mean for age) (4). Silver–Russel syndrome was ruled out by standard genetic investigations (4). At the age of 6.2 years, a comparative genomic hybridization (CGH) array unveiled a 17q22 deletion of maternal origin (chr17: 57,086,110-57,229,241 [Hg19]), involving a region including *TRIM37* (4); DNA Sanger sequencing identified a novel *TRIM37* variant, c.1949-12A>G in intron 18, of paternal origin, confirming MUL syndrome (4). At the age of 8, an echocardiogram revealed an atrial septal defect, bi-atrial dilatation, and constrictive pericarditis treated with furosemide and spironolactone. During the follow up, the patient developed severe spleen and liver enlargement with steatosis and multiple cystic lesions. A transient increase of serum levels of liver enzymes, gamma-glutamyl transferase (GGT), and bile acids was also detected. Ultrasound-based transient elastography (TE), performed to assess liver fibrosis, showed an increased liver stiffness, ranging from 19.5 to 21.9 kPa. Total proteins, albumin, bilirubin, platelets, and hemoglobin were in the normal range. Renal and pancreatic cystic lesions were also detected. Magnetic resonance imaging (MRI) documented hypoplasia of the adenohypophysis, mega cisterna magna, arachnoid cyst of the right temporal lobe, and syringomyelia, extended between D8 and D12. At the age of 8.6, the child was admitted to our Pediatric Department due to fever, cough, and dyspnea. Chest high-resolution computed tomography (HRCT) revealed an interstitial lung disease characterized by widespread thickening of the intralobular interstitium and the thickened appearance of the interlobular septa of the basal territories. Serological investigations ruled out *C. pneumoniae*, adenovirus, cytomegalovirus, and human immunodeficiency virus infections. Sputum culture was positive for *St. Aureus* and *H. influenza*. Lung functional tests showed a restrictive pattern persisting over time.

An immunological examination, performed at the age of 9.6, revealed IgG levels (IgG 202 mg/dL) well below the sex-age predetermined reference ranges (12), requiring intravenous immunoglobulins treatment; however, normal IgG titers against rubella and mumps virus were detected. Normal IgA (73 mg/dL), IgM (90 mg/dL), and a slight increase of IgE levels (446 IU/mL) were reported.

**Figure 1:**
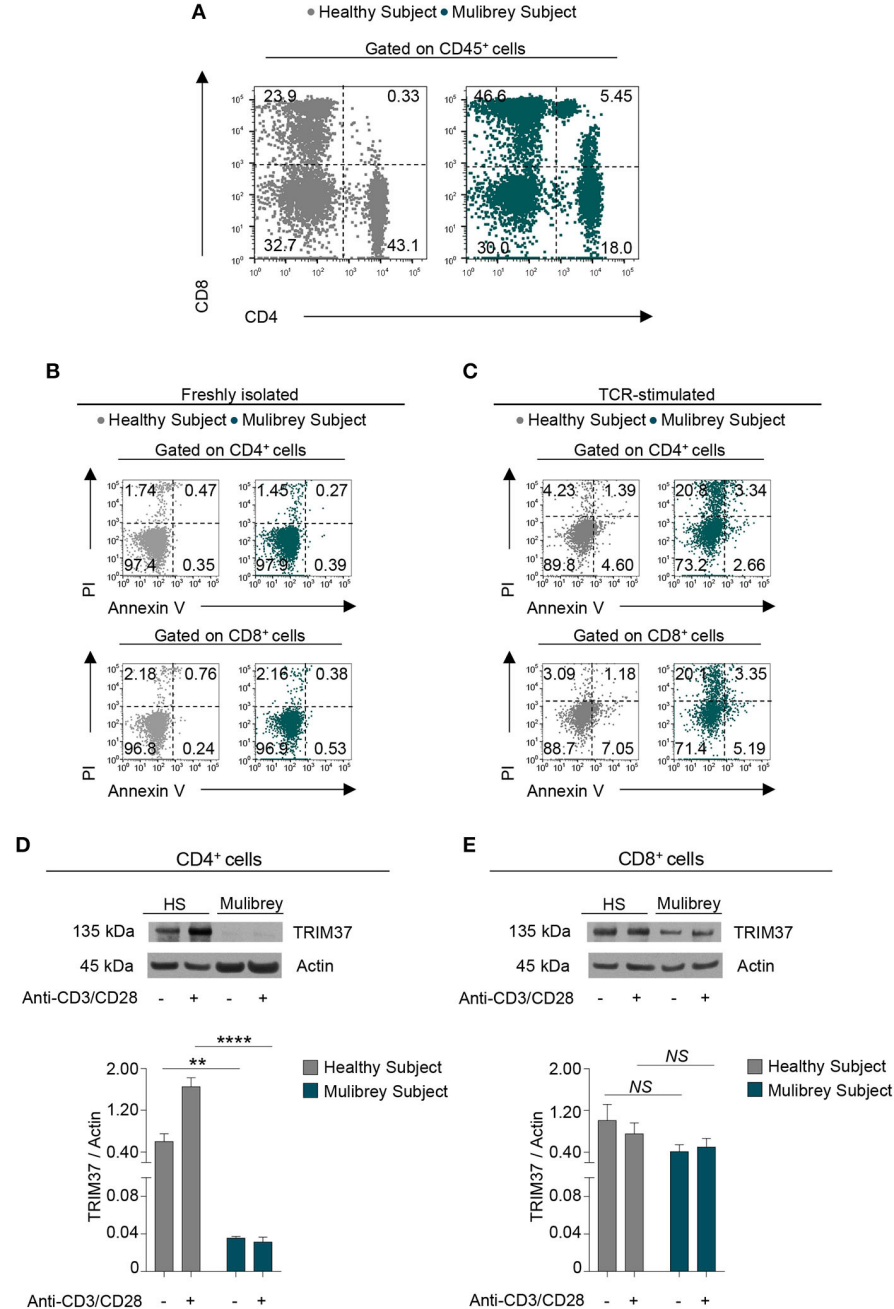
CD4^+^ T cells from the MUL child are reduced in number and expressed low levels of intracellular TRIM37. **(A)** Flow cytometry plots show CD4^+^ and CD8^+^ cell frequency in peripheral blood mononuclear cells (PBMCs) from the MUL child and matched healthy control. Numbers in plots indicate positive cells. Data are from one representative experiment out of six. **(B)** Flow cytometry plots show the Annexin V and Propidium Iodide (PI) staining in CD4^+^ (upper panels) and CD8^+^ (lower panels) T cells from the MUL and healthy child. Numbers in plots indicate positive cells. Data are from one representative experiment out of three. **(C)** Flow cytometry plots show Annexin V and PI in 48 h TCR-stimulated CD4^+^ (upper panels) and CD8^+^ (lower panels) T cells from the MUL and healthy child. Numbers in plots indicate positive cells. Data are from one representative experiment out of two. **(D)** The upper panel shows representative immunoblot for TRIM37 and actin proteins in unstimulated and 30 min TCR-stimulated CD4^+^ T cells from the MUL and healthy child. The lower panel shows the relative densitometric quantitation of the TRIM37 protein normalized on actin in CD4^+^ T cells from the MUL and healthy child, in the aforementioned experimental conditions. Data are shown as mean ± SEM (*n* = 4 densitometric quantitation derived from four films with different exposure timing). **P < 0.01; ****P < 0.0001 by unpaired two-tailed ordinary 2-way ANOVA corrected with Bonferroni's multiple comparisons test. **(E)** The upper panel shows representative immunoblot for TRIM37 and actin proteins in unstimulated and 30 min TCR-stimulated CD8^+^ T cells from the MUL and healthy child. The lower panel shows the relative densitometric quantitation of TRIM37 protein normalized on actin in CD8^+^ T cells from the MUL and healthy child, in the aforementioned experimental conditions. Data are shown as mean ± SEM (*n* = 4 densitometric quantitation from four films with different exposure timing). *NS*, not significant by unpaired two-tailed ordinary 2-way ANOVA corrected with Bonferroni's multiple comparisons test.

Analysis of circulating immune cell populations unveiled that the MUL subject had an increased number of neutrophils, basophils, and monocytes compared with sex-age matched healthy controls (Table 1). Further, a marked lymphopenia, ranging from 672 to 1958 cell/mm^3^, was also observed in the MUL case (Table 1). More in detail, the MUL child showed a significant reduction of circulating CD4^+^ T cells compared with sex-age matched healthy subjects (Table 1 and Figure 1A). The MUL child also displayed an increased frequency of both CD8^+^ T cells and double positive CD4^+^CD8^+^ T lymphocytes in peripheral blood (Table 1 and Figure 1A). No significant differences were observed in the other main lymphocyte subset, such as Natural Killer (NK) and B cells (Table 1). Finally, the MUL child displayed a strong reduction of proliferating CD4^+^CD28^+^ cells and an increase of CD8^+^CD11b^+^ cells, an heterogeneous population precursor of lymphokine activated killer (LAK) cells (Table 1) (13).

**Table 1:** Absolute numbers and percentages (in brackets) of circulating immune cells in the Mulibrey patient and sex-age related healthy children (*n* = 9).

**Immune cells**	**Healthy Children (*n* = 9; age-sex matched)**	**Mulibrey Patient (at 8, 9,10 years)**	***P*-value**
Leucocytes	5308.89 ± 1925.60	6071.67 ± 2221.20	*NS*
Neutrophils	3359 ± 1715 (53.36% ± 6.93)	3468 ± 1332 (61.78%^*^± 5.26)	^*^P < 0.05
Eosinophils	166.2 ± 109.1 (2.84% ± 1.76)	106.7 ± 48.85 (1.9% ± 0.54)	*NS*
Basophils	24.51 ± 10.74 (0.42% ± 0.17)	105^*^± 78.17 (1.97% ± 1.70)	^*^P < 0.05
Monocytes	290 ± 162.8 (4.85% ± 1.61)	523.3^*^± 159.7 (9.63%^**^± 2.02)	^*^P < 0.05 ^**^P < 0.01
Lymphocytes	2264.22 ± 524.08 (45.19% ± 12.32)	1341.17^**^± 456.54 (23.58%^**^± 8.18)	^**^P < 0.01
CD3^+^ cells	1591.22 ± 377.36 (70.56% ± 6.11)	955.5^**^± 405.28 (69.17% ± 9.56)	^**^P < 0.01
CD4^+^ cells	928.33 ± 228.93 (41.22% ± 6.42)	231^****^± 123.96 (16.33% ^****^± 4.46)	^****^P < 0.0001
CD8^+^ cells	510.94 ± 194.42 (22.33% ± 4.53)	652 ± 262.72 (47.33%^****^± 6.89)	^****^P < 0.001
CD4^+^CD8^+^ cells	20.72 ± 15.11 (1.02% ± 0.87)	79.02^**^± 37.33 (5.79%^***^± 1.39)	^**^P < 0.01 ^***^P < 0.001
NK cells	218.44 ± 147.22 (9.22% ± 4.84)	108.33 ± 54.70 (8.5% ± 4.42)	*NS*
CD19^+^ cells	363.08 ± 121.99 (16.56% ± 5.75)	268.5 ± 87.27 (21.5% ± 8.89)	*NS*
CD3^+^CD16^+^CD56^+^ cells	64.22 ± 42.94 (3.01% ± 2.03)	73.74 ±40.98 (5.43% ± 2.64)	*NS*
CD4^+^CD28^+^ cells	861.7 ± 204.12 (38.29% ± 5.35)	194.52 ^****^*± 103.75 (14%^****^± 3.68)	^****^P < 0.0001
CD8^+^CD11b^+^ cells	78.7 ± 71.81 (3.45% ± 2.88)	213.33^*^± 155.71 (16.33%^**^± 11.78)	^*^P < 0.05 ^**^P < 0.01

*Data are expressed as mean ± SD. The absolute number per mm^3^ of lymphocyte populations was calculated as follows: percent of a given cell population multiplied by the number of lymphocytes/100*.

* *P < 0.05*,

** *P < 0.01*,

*** *P < 0.001*,

**** *P < 0.0001 by two-tailed parametric Student's t-test. NS, not significant*.

A thymic MRI was performed in order to evaluate parenchymal structural abnormalities, including fibrosis, and no alterations were found.

### Reduced Frequency of CD4^+^ Lymphocytes Associated With Their Low TRIM37 Expression in the MUL Child

To assess whether the low number of circulating CD4^+^ cells observed in the MUL child could be due to an increase rate of their cell death, Annexin V and Propidium staining was performed. *Ex vivo* flow cytometry analysis revealed that the freshly isolated CD4^+^ and CD8^+^ cells from the MUL child showed a negligible rate of cell death as compared with the healthy control (Figure 1B). Upon T cell receptor (TCR) stimulation, both CD4^+^ and CD8^+^ lymphocytes from the MUL child displayed a higher rate of cell death than the healthy child (Figure 1C); however, no differences in cell death were observed between activated CD4^+^ and CD8^+^ cells from the MUL subject (Figure 1C), suggesting that the selective reduction of CD4^+^ lymphocytes was not ascribed to an increase of their cell death.

Next, to evaluate the presence of the TRIM37 protein in T lymphocyte subsets, we analyzed its expression in both CD4^+^ and CD8^+^ T cells, isolated from peripheral blood mononuclear cells (PBMCs) of the MUL and the sex-age related healthy child. Western Blot analysis revealed that unstimulated CD4^+^ T cells from the MUL child expressed a negligible amount of the TRIM37 protein compared with the healthy child (Figure 1D). Strikingly, upon TCR engagement CD4^+^ cells from the MUL subject did not increase intracellular levels of TRIM37 as compared to control CD4^+^ lymphocytes (Figure 1D). No significant differences were observed in TRIM37 levels, in both unstimulated and TCR-stimulated CD8^+^ T cells, between the MUL and healthy child (Figure 1E); notably, higher levels of TRIM37 expression were observed in CD8^+^ rather than in CD4^+^ T cells from the MUL patient (Figures 1D,E). These findings reveal a selective reduction of TRIM37 levels in CD4^+^ T cells from the MUL child.

### Differentiation and Activation Status of Peripheral T Cells From the MUL Child

**Figure 2:**
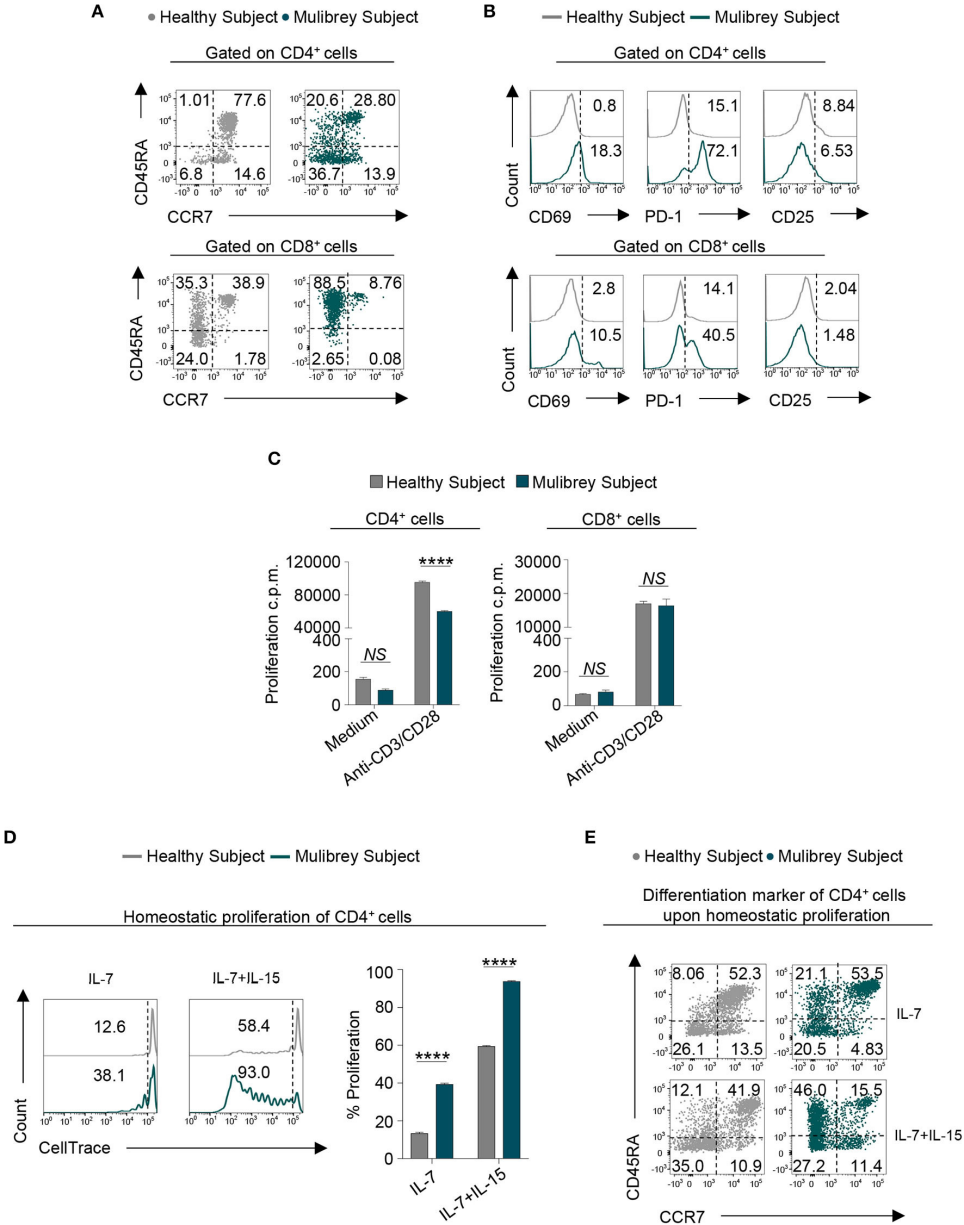
Altered phenotype and proliferation capability in CD4^+^ T cells from the MUL child. **(A)** Flow cytometry plots indicating T cell differentiation markers of both CD4^+^ (upper panels) and CD8^+^ (lower panels) lymphocytes in the MUL and healthy child, respectively. Based on the expression of CD45RA and CCR7 molecules, cells were distinct in naïve (CD45RA^+^CCR7^+^), central memory (CD45RA^−^CCR7^+^), effector memory (CD45RA^−^CCR7^−^), and terminally differentiated effector memory (CD45RA^+^CCR7^−^). Numbers in plots indicate positive cells. Data are from one representative experiment out of three. **(B)** Flow cytometry histograms show the percentage of several activation markers, including CD69, PD-1, and CD25, in both CD4^+^ (upper panels) and CD8^+^ (lower panels) lymphocytes from the MUL and healthy child. Numbers in plots indicate positive cells. Data are from one representative experiment out of three. **(C)** Column graphs indicate the proliferation counts per minute (c.p.m.), measured as thymidine incorporation, of the CD4^+^ (left panel) and CD8^+^ (right panel) lymphocytes in the presence or absence of anti-CD3/CD28 stimulation, from the MUL and healthy child, respectively. Data are expressed as mean ± SEM (*n* = 3). ****P < 0.0001 by unpaired two-tailed ordinary 2-way ANOVA corrected with Bonferroni's multiple comparisons test. *NS*=not significant. **(D)** Flow cytometry plots (left panels) show homeostatic proliferation of freshly isolated CD4^+^ T cells cultured for 9 days in the presence of either IL-7 or IL-7 plus IL-15. Numbers in plots indicate positive cells. Data are from one representative experiment out of three. The column graph (right panel) indicates cumulative data of homeostatic proliferation in the aforementioned experimental conditions. Data are expressed as mean ± SEM (*n* = 3). ****P < 0.0001 by unpaired two-tailed ordinary 2-way ANOVA corrected with Bonferroni's multiple comparisons test. **(E)** Flow cytometry plots indicate T cell differentiation markers of CD4^+^ lymphocytes in the MUL and healthy child upon *in vitro* homeostatic proliferation in the aforementioned experimental conditions. Based on the expression of CD45RA and CCR7 molecules, cells were distinct in naïve central memory, effector memory, and terminally differentiated effector memory. Numbers in plots indicate positive cells. Data are from one representative experiment out of three.

To investigate whether the observed TRIM37 mutations in our MUL case could affect T cell activation and differentiation status, we performed a multiparametric flow cytometry analysis, of both circulating CD4^+^ and CD8^+^ T cells. *Ex vivo* analysis revealed an enrichment of CD4^+^ T cells with both an effector memory (CD45RA^−^CCR7^−^) and terminally differentiated effector memory (CD45RA^+^CCR7^−^) phenotype in the MUL child, compared with the sex-age related healthy child (Figure 2A, upper panels). Strikingly, large amount of CD8^+^ T cells from the MUL patient showed a terminally differentiated effector memory (CD45RA^+^CCR7^−^) phenotype (Figure 2A, lower panels). As a consequence, a reduction of naïve cells was observed in both CD4^+^ and CD8^+^ cell compartments in the MUL child (Figure 2A). Analysis of surface markers revealed that both T cell subsets from the MUL child expressed higher levels of canonical activation molecules, including CD69 and PD-1, as compared to the healthy subject (Figure 2B). Whereas, no differences were observed in the expression of CD25 molecule on both CD4^+^ and CD8^+^ T lymphocytes between the MUL and healthy subject (Figure 2B). Together, these data suggest that TRIM37 alterations coupled with a T cell memory-like phenotype in the MUL child.

### Proliferative Ability and Homeostatic Proliferation of CD4^+^ T Lymphocytes in the MUL Child

Next, we tested whether the lower frequency of circulating CD4^+^ cells observed in the MUL child could be due to a defect in their proliferative response. We found that compared to healthy control, TCR-stimulated CD4^+^ cells from the MUL child showed a significant reduction in their proliferative capability, measured as thymidine incorporation (Figure 2C, left panel); no proliferative defects were observed in TCR-activated CD8^+^ lymphocytes from the MUL child (Figure 2C, right panel).

To gain more insight on CD4^+^ T cell proliferation, we evaluated their proliferative response upon homeostatic stimuli. To this end, CD4^+^ T cells were cultured for 9 days in the presence of either interleukin (IL)-7 or IL-7 plus IL-15. Flow cytometric analysis unveiled that in both experimental conditions, CD4^+^ cells from the MUL subject displayed a stronger homeostatic proliferation than CD4^+^ cells from healthy control (Figure 2D). During homeostatic proliferation, the majority of CD4^+^ lymphocytes from the MUL child acquired a memory phenotype, as testified by the high frequency of the terminally differentiated effector memory cells re-expressing CD45RA (TEMRA) subset (Figure 2E).

All together, these data indicate that CD4^+^ T cells from the MUL patient are unable to proliferate in response to TCR stimulation, but they are more sensitive to homeostatic stimuli.

### Activation Markers and Cytokine Production in TCR-Stimulated Lymphocytes in the MUL Child

**Figure 3:**
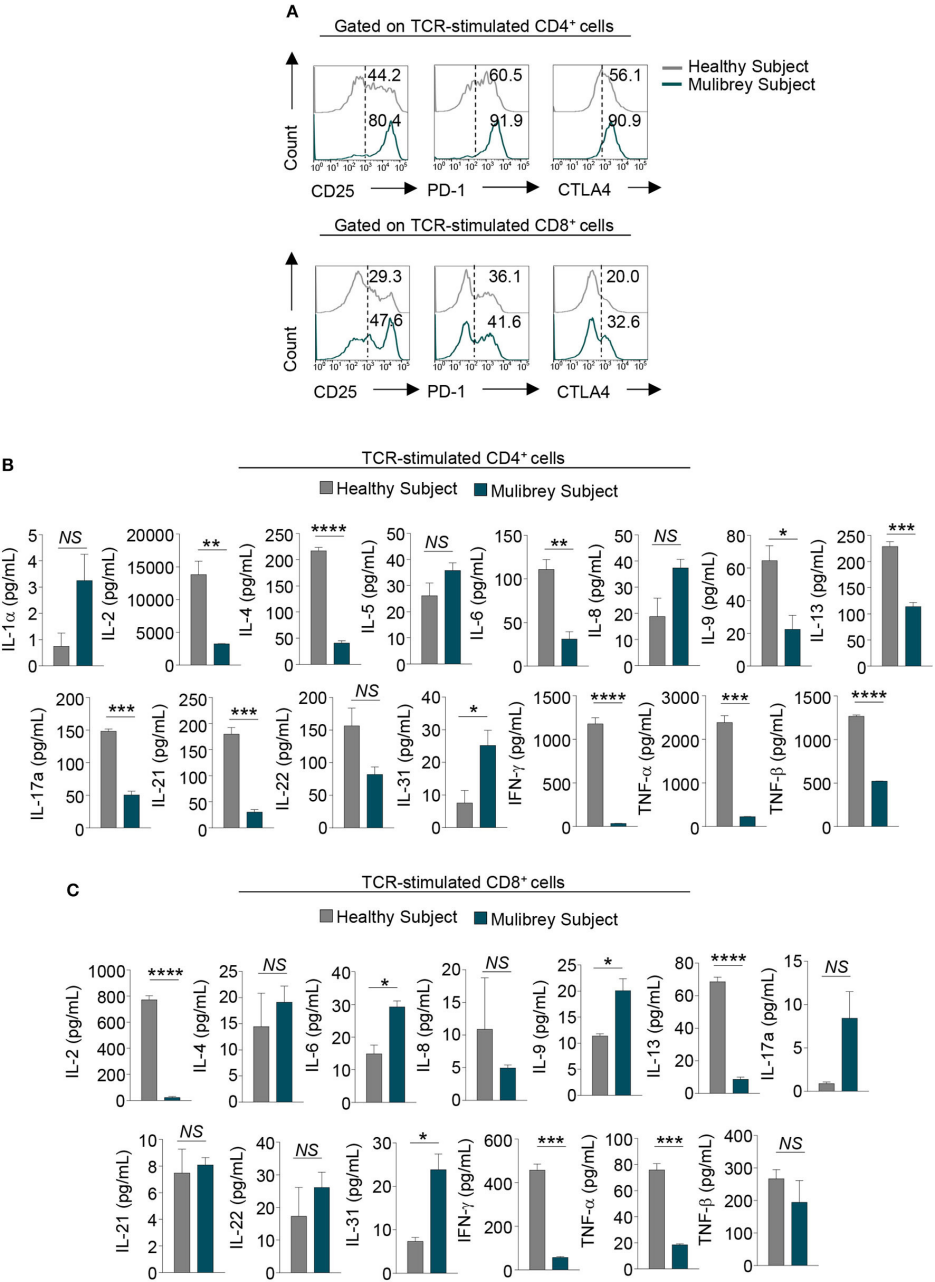
Impaired activation and cytokine production in CD4^+^ T cells from the MUL child. **(A)** Flow cytometry histograms show the percentage of several activation markers, including CD25, PD-1, and CTLA-4, in 48 h TCR-stimulated CD4^+^ (upper panels) and CD8^+^ (lower panels) lymphocytes from the MUL and healthy child. Numbers in plots indicate positive cells. Data are from one representative experiment out of two. **(B)** Column graphs show cytokines released by CD4^+^ (upper panels) and **(C)** CD8^+^ lymphocytes upon 48 h TCR-activation, from the MUL and healthy child. Data are expressed as mean ± SEM (*n* = 3). *P < 0.05; **P < 0.01; ***P < 0.001; ****P < 0.0001 by unpaired two-tailed Student's *t*-test. *NS*, not significant.

To characterize more in depth T lymphocyte subsets from the MUL child, we evaluated the expression of functional/activation markers and cytokine production *in vitro* upon TCR-activation. Flow cytometry analysis revealed that both CD4^+^ and CD8^+^ T cells from the MUL child expressed higher levels of the main activation markers, including CD25, PD-1, and CTLA-4 as compared with the healthy child (Figure 3A); of note, upon TCR-engagement, the levels of such molecules were more evident in CD4^+^ rather than CD8^+^ T cells from the MUL patient (Figure 3A).

Multiplex analysis of cytokine production in supernatant of TCR-stimulated CD4^+^ and CD8^+^ T lymphocytes revealed that both cell subsets from the MUL child had a cytokine profile different from that observed in the sex-age matched control. Specifically, CD4^+^ lymphocytes from the MUL child secreted significantly lower levels of IL-2, IL-4, IL-6, IL-9, IL-13, IL-17A, IL-21, IL-22, interferon (IFN)-γ, tumor necrosis factor (TNF)-α, and TNF-β (Figure 3B); on the contrary, CD4^+^ cells produced higher levels of IL-1α, IL-5 and IL-8, and IL-31 (Figure 3B). Moreover, CD8^+^ lymphocytes produced lower levels of IL-2, IL-8, IL-13, IFN-γ, TNF-α, and TNF-β (Figure 3C); while they secreted higher amounts of IL-4, IL-6, IL-9, IL-17A, IL-22, and IL-31, as compared to the healthy child (Figure 3C).

Taken together, these findings indicate that T cells from the MUL patient exhibit a dysregulated *in vitro* activation and are functionally impaired in terms of cytokine production.

## Discussion

Here we reported that specific *TRIM37* mutations (4) detected in our MUL case were associated with an impairment in both frequency and proliferative ability of the CD4^+^ T cell subset. No differences were observed in the proliferative response of freshly isolated CD8^+^ T lymphocytes from the same MUL child. These findings are in line with our biochemical experiments revealing that TRIM37 protein reduction was highly extended in CD4^+^ compared to CD8^+^ lymphocytes in the MUL patient. However, both CD4^+^ and CD8^+^ T lymphocytes from the patient showed a terminally differentiated memory phenotype, unusual in age-matched healthy children (11). Hereto, the only immunological defect observed in a girl with MUL syndrome was the subnormal concentration of serum IgG associated with a reduced B cell number (9).

Although it has been shown that the TRIM family was involved in innate immune response (14), none of the published evidence associated T lymphocyte derangement with *TRIM37* mutations in MUL syndrome (5, 10). It is likely that the specific *TRIM37* mutations present in our MUL case (4) encoded for a defective TRIM37 protein, altering its conformation, cellular localization, and protein-protein interaction. As a consequence, defective TRIM37 could affect signaling pathways controlling T cell proliferation and differentiation status in the presence of both homeostatic stimuli and TCR-engagement. Indeed, following T cell stimulation, intracellular programs work together with stop signals to restore quiescent status; these coordinated events are crucial for pathogen clearance, resolution of immune response, and for maintaining peripheral T cell pool (15, 16). Thus, defects in the regulation of activation pathways may lead to the development of several pathological conditions (17).

Protein ubiquitination is one of the most important mechanisms used to regulate signal transduction pathways in the immune cell population, including T lymphocytes (18). Indeed, it has been reported that deranged ubiquitination processes may alter T cell activation, as well as their differentiation, cytokine production, and cell cycle progression (19, 20). For example, TRIM28-deficient T cells show impaired proliferation and IL-2 production; while TRIM33 deletion reduced pro-inflammatory T helper (Th)17 cells and ameliorated experimental autoimmune encephalomyelitis (EAE) (21–23).

Our data may fit in the framework where E3 ubiquitin ligase enzymes, including the TRIM superfamily proteins, are crucial constituents to turn-off activation signaling and to start protein degradation processes, especially in CD4^+^ T cells (24, 25). It is possible that the altered TRIM37 protein in MUL CD4^+^ cells may fail to down-regulate the activation process, as testified by increased activation markers expressed on MUL CD4^+^ T cells. This is also in agreement with the *ex vivo* phenotype of MUL CD4^+^ cells, which displayed a more differentiated/activated status. Indeed, it is possible that in response to homeostatic stimuli, during lifetime, naïve CD4^+^ T cells of the MUL child expand and differentiate into memory T cells. Hence, we hypothesize that deranged stop signaling (due to defective TRIM37) enhances homeostatic proliferation of naïve CD4^+^ T cells, leading to their rapid differentiation in memory lymphocytes which are characterized by reduced proliferation in response to TCR (26). In this context, it has been shown that repeated cycles of proliferation determine the disappearance of cell-surface CD28 molecules (27), suggesting this as the basis of CD4^+^CD28^+^ cell reduction observed in the MUL case.

We also noticed that, except for pneumonia, our MUL case had a relatively low rate of infectious diseases during his life, despite the CD4^+^ cell deficit. This apparent paradox could be explained by recent observations showing that memory CD4^+^ T cells are strongly protective against infections, and act in synergy with CD8^+^ T lymphocytes in viral clearance (28).

The major limitation of this study relies on the absence of mechanistic insights linking *TRIM37* specific mutations with T cell fate. *In vitro* and *in vivo* approaches (RNA interference, CRISPR-CAS9, or knock-out mice models) targeting TRIM37 will be helpful to directly elucidate the role of this protein in the T cell development and function, particularly in CD4^+^ lymphocytes.

In conclusion, our study identified the TRIM37 protein as a potential new player involved in the differentiation and functional activity of T lymphocytes. These findings may open the way for additional research to explore in depth the role of TRIM37 in the immune response both in health and immune-mediated disorders.

## Methods

### Patient and Controls

The MUL patient and sex-age related healthy subjects were recruited at Dipartimento di Scienze Mediche Traslazionali, Sezione di Pediatria, University of Naples “Federico II.” Blood samples from the MUL subject and from healthy individuals were withdrawn at 8.00 a.m. into heparinized Vacutainers and processed within the following 4 h.

### Flow Cytometry and Cell Isolation

Immune cell profiling from the MUL and healthy subjects was done at the time of blood drawing. Whole blood cells were analyzed with a clinical-grade hemocytometer to determine absolute lymphocyte numbers in each sample and 100 μl of blood was incubated for 30 min at room temperature with specific combinations of human monoclonal antibodies as previously described (29).

For the simultaneous evaluation of molecules involved in differentiation and activation status, PBMCs from the MUL and healthy children were isolated by stratifying heparinized whole blood on Ficoll-Hypaque (GE Healthcare) and then stained with the following antibodies: FITC-anti-CD45RA (clone REA562, Miltenyi Biotec), BB700-anti-CCR7 (clone 3D12, BD Horizon), APC-anti-CD3 (clone UCHT1, BD Pharmingen), APC-H7-anti-CD4 (clone RPA-T4, BD Pharmingen), PE-Cy7- or BV421-anti-CD8 (clone RPA-T8, BD Pharmingen), PercyP-Cy5.5-anti-CD69 (clone FN50, BD Pharmingen), PE-Cy7-anti-CD25 (clone M-A251, BD Pharmingen), APC-anti-CD152/CTLA-4 (clone BN13, BD Pharmingen), and BV421-anti-PD-1 (clone EH12-1, BD Horizon). Staining for intracellular factors was performed using fixation and a permeabilization FoxP3 buffer kit (BD Pharmingen), according to the manufacturer's instructions.

For the evaluation of cell death, PBMCs were stained with the following antibodies: FITC anti-Annexin V (BD Pharmingen), PE-Cy7 anti-CD8 (clone RPA-T8, BD Pharmingen), APC anti-CD3 (clone UCHT1, BD Pharmingen), BV421 anti-CD4 (clone RPA-T4, BD Horizon), and Propidium Iodide (BD Pharmingen); Annexin V buffer (BD Pharmingen) was used for the staining according to the manufacturer' instructions.

Samples were acquired using a FACSCanto II (BD Bioscience) and cytofluorimetric analyses were performed using FlowJo Software (FlowJo, LLC) as described (30).

### Western Blotting

To perform a Western blotting assay, CD4^+^ and CD8^+^ cells were purified (90–95% purity) from PBMCs by magnetic cell separation with a CD4^+^ T Cell Kit and a CD8^+^ T Cell Kit (both from Invitrogen, Thermo Fisher Scientific). Cells were cultured in round-bottom 96-well plates (Falcon, Becton Dickinson) with RPMI-1640 medium (Gibco, Thermo Fisher Scientific) supplemented with 5% autologous plasma in the presence or absence of anti-CD3/CD28–coated Dynabeads (0.2 beads/cell; Gibco, Thermo Fisher Scientific) for 30 min. Unstimulated and TCR-stimulated CD4^+^ and CD8^+^ cells were lysed on ice in RIPA buffer (Sigma-Aldrich) plus SIGMAFAST Protease Inhibitor (Sigma-Aldrich), and Sigma Phosphatase Inhibitor (Sigma-Aldrich) for 20 min. Protein concentration was calculated by a BCA protein assay kit (Pierce, Thermo Fisher Scientific) and 15 μg of proteins were separated by SDS-PAGE under reducing conditions, as previously described (31). Membrane was incubated overnight at 4°C with anti-TRIM37 (clone D7U2L, Cell Signaling). Then, the filter was washed three times in phosphate-buffered saline 0.5% Tween 20 (PBST) and incubated with a peroxidase-conjugated secondary antibody (GE Healthcare) for 1 h. After washing with PBST, peroxidase activity was detected with the ECL system (Roche) or Femto (Pierce, Thermo Fisher Scientific). We scanned four films with different timing exposures for TRIM37. All signals were quantified normalizing to actin (clone C4, Santa Cruz Biotechnolgy). Densitometric analysis was performed using ImageJ Software (NIH).

### Proliferation Assay

CD4^+^ and CD8^+^ cells were purified as described above. Cells were cultured (2.5 × 10^4^ cells/well) in round-bottom 96-well plates (Falcon, Becton Dickinson) with RPMI-1640 medium (Gibco, Thermo Fisher Scientific) supplemented with 5% autologous plasma in the presence or absence of anti-CD3/CD28–coated Dynabeads (0.1 beads/cell; Gibco, Thermo Fisher Scientific) for 60 h. After 48 h, supernatant were collected and stored at −20°C until use and then [^3^H]thymidine (0.5 μCi/well; Amersham-Pharmacia Biotech) was added to cell cultures; cells were harvested 12 h later. Radioactivity was measured with a β-cell plate scintillation counter (Wallac).

### Homeostatic Proliferation Assay

CD4^+^ cells were purified as described above and were labeled with the fluorescent dye CellTrace Violet (Invitrogen, Thermo Fisher Scientific). Cells were cultured (5 × 10^4^ cells/well) in round-bottom 96-well plates (Falcon, Becton Dickinson) with RPMI-1640 medium (Gibco, Thermo Fisher Scientific) supplemented with 5% autologous plasma in the presence of either human recombinant (hr) IL-7 (25 ng/mL; R&D) or hrIL-7 plus hrIL-15 (25 ng/mL; R&D). After 9 days, cells were stained to evaluate differentiation status, as described above. Samples were acquired by using a FACSCanto II (BD Bioscience) to evaluate homeostatic proliferation and marker of differentiation.

### T Cell Activation and Cytokine Assessment

To evaluate activation molecules and cell death upon TCR-engagement, 5 × 10^5^ PBMCs were cultured in 96-well round-bottom plates (Falcon, Becton Dickinson) with RPMI-1640 medium (Gibco, Thermo Fisher Scientific) supplemented with 5% autologous plasma in the presence of anti-CD3 (0.1 μg/mL; clone OKT3) for 48 h. After 48 h, flow cytometry staining was performed with afore-mentioned antibodies.

Cytokine production was analyzed from supernatant of CD4^+^ and CD8^+^ cells, collected as described above, using the bead-based multianalyte immunoassay (Invitrogen, Thermo Fisher Scientific) according to the manufacturer's recommendations, and measured by Multiplex technology (Luminex 200, Luminex). xPONENT 3.1 software (Luminex) was used for data acquisition.

### Statistical Analysis

Statistical analyses were carried out by the GraphPad Prism software (GraphPad, California, USA). Comparisons were performed by unpaired two-tailed Student's *t*-test or ordinary 2-way ANOVA corrected with Bonferroni's multiple comparisons test, with *P* < 0.05 values denoting statistical significance.

## Data Availability Statement

The raw data supporting the conclusions of this article will be made available by the authors, without undue reservation.

## Ethics Statement

The studies involving human participants were reviewed and approved by Comitato Etico Federico II Università degli Studi di Napoli—Federico II. Written informed consent to participate in this study was provided by the participants' legal guardian/next of kin. Written informed consent was obtained from the minor(s)' legal guardian/next of kin for the publication of any potentially identifiable images or data included in this article.

## Author Contributions

SB performed most of the experiments and data analyses. ML and LM performed Western Blot experiments. FG, CPa, and FP performed immunophenotype experiments and data analyses. EM, VF, EC, RP, and GG collected clinical data and provided human samples. SB, CPr, and EM analyzed the data and interpreted the results. SB, AF, and CPr were involved in the discussion about the data. GM and MB helped with critical revision of the work for its intellectual content. AF, CPi, and MG designed the study and wrote the manuscript. All authors contributed to the article and approved the submitted version.

## Conflict of Interest

The authors declare that the research was conducted in the absence of any commercial or financial relationships that could be construed as a potential conflict of interest.
